# Association Between a Hybrid Video-Guided Multicomponent Exercise Program With Staff Assistance and Free-Living Gait in Older Adults With Knee and Low Back Pain: Secondary Analysis of a Randomized Controlled Trial

**DOI:** 10.2196/97414

**Published:** 2026-10-08

**Authors:** Shuichi P Obuchi, Tomoyuki Arai, Hideaki Ishibashi, Takashi Amari, Hisashi Kawai

**Affiliations:** 1 Research Team for Human Care, Tokyo Metropolitan Institute for Geriatrics and Gerontology, 35-2 Sakae-cho, Itabashi-Ku, Tokyo, 1730015, Japan, 81 3-3964-3241 ext 4243, 81 3-3964-1844; 2 Faculty of Health Sciences, Saitama Medical University, Saitama, Japan; 3 Ina Hospital, Saitama, Japan

**Keywords:** hybrid video-guided multicomponent exercise program, randomized controlled trial, gait, active energy expenditure, pain

## Abstract

**Background:**

Older adults with chronic joint pain often exhibit decreased mobility. Daily walking measures are being increasingly used in aging research as objective indicators of mobility.

**Objective:**

In this study, we aimed to examine the effects of a 12-week hybrid exercise-based program on daily-life walking characteristics.

**Methods:**

A total of 59 community-dwelling older adults (aged ≥65 years) with mild to moderate knee and/or low back pain were randomized to either a hybrid exercise group or an educational control group. Daily-life walking was monitored using an ankle sensor. Daily observation data were collected within 2 planned 7-day windows. Primary parameters included activity volume and gait characteristics. Statistical analyses were conducted based on a modified intention-to-treat principle using linear mixed-effects models, with a per-protocol set used for sensitivity analyses.

**Results:**

The mixed-effects analysis based on the modified intention-to-treat principle (n=52 participants; 696 observations) suggested an effect of the intervention on improving walking quality, particularly step length (β=2.39 cm; *P*=.04; Hedges *g*=0.493), with a smaller effect with borderline statistical significance observed on improving walking speed (β=0.05 m/s; *P*=.06; Hedges *g*=0.496). Cadence, daily distance, daily steps, active energy expenditure, and accumulated 3 or more metabolic equivalent of task activities showed no clear difference between the groups. In the per-protocol sensitivity analysis (n=44), the same directional pattern of improvement was observed for step length (β=1.95 cm; *P*=.13; Hedges *g*=0.365) and walking speed (β=0.04 m/s; *P*=.14; Hedges *g*=0.368); however, the estimates were imprecise.

**Conclusions:**

A 12-week hybrid exercise program may improve the quality of daily-life walking, with the clearest effect observed on step length. Given the attenuation after multiplicity-aware interpretation and in sensitivity analyses, these findings should be interpreted as suggestive rather than definitive.

## Introduction

Walking function maintenance is a key target for preserving independence in older adults because gait-related measures such as walking speed have been reported as early and strong predictors of subsequent decline in instrumental activities of daily living among community-dwelling older adults [1]. Musculoskeletal pain is a major barrier to maintaining walking function in older adults [2,3]. In Japan, approximately 42.2 million adults (41.2%) experience musculoskeletal pain, with 9.1 million (8.8%) reporting that the pain interferes with their daily activities [4]. The national burden of low back, hip, and knee pain is projected to increase up to 2055 [5]. These burdens are particularly pronounced in older adults. In Japan’s population-based ROAD (Research on Osteoarthritis Against Disability) study of community-dwelling people aged 60 years and older, the prevalences of radiographic knee osteoarthritis (Kellgren-Lawrence grade≥2) were 47.0% and 70.2% in men and women, respectively [6]. In the related LOCOMO (Longitudinal Cohorts of Motor System Organ) study, the prevalence of knee pain was 32.7%, and that of lumbar pain was 37.7%; overall, 12.2% of the participants reported both knee and low back pain concurrently [7]—the very combination targeted by the present study. Scalable approaches to musculoskeletal pain are therefore urgently needed to prevent pain-related functional decline in aging societies.

Numerous randomized controlled trials have demonstrated the effectiveness of multicomponent exercise programs in relieving symptoms and improving function in individuals with chronic knee and low back pain. For knee pain, programs combining stretching, strengthening, and aerobic exercises improve pain and physical function [8]. For low back pain, similar improvements require more than exercise programs alone; interventions integrating exercise with pain coping and behavioral skill training have shown sustained benefits [9-11]. Although many of these interventions were delivered under professional supervision, the high prevalence of knee and back pain in the older adult population highlights the need for more accessible exercise programs [12].

To improve access, audiovisual programs and remote digital interventions have been evaluated for musculoskeletal pain management. These include internet-delivered exercise programs with pain coping skill training for chronic knee pain and digital care programs for chronic low back pain [13,14]. However, adherence to home exercise programs declines over time. Key barriers to sustained participation include symptom flare-ups, fear of worsening pain, limited feedback or support outside of supervised settings, and difficulty integrating exercise into daily routines despite the flexibility of video-based delivery [15-17]. These practical constraints suggest that a hybrid program in which participants perform video-guided exercises during on-site sessions with staff support may be more effective.

Daily walking measures obtained in real-world settings are increasingly used in aging research and complement laboratory-based assessments as objective indicators of mobility [18-20]. Reference values for daily-life walking parameters have been reported in the Japanese population [21], and daily-life walking speed, as assessed using smartphones, has been used to evaluate frailty [22]. Daily-life walking speed is increasingly used as an objective indicator of mobility, disability, and other adverse health outcomes in older adults [23,24].

Thus, in this secondary analysis of data from a randomized controlled trial, we aimed to examine whether a hybrid exercise-based intervention improved objectively assessed daily-life walking outcomes. In the parent randomized controlled trial, participants were allocated to either a hybrid multicomponent exercise program (video-guided exercises with staff assistance during twice-weekly on-site sessions) or a once-a-week face-to-face class. Primary symptom outcomes will be reported separately. We also sought to compare the effects of the two programs on objectively assessed daily-life walking outcomes.

## Methods

### Study Design and Setting

This study involved a secondary analysis of data from a randomized controlled trial that evaluated a hybrid, exercise-based program targeting knee and low back pain. Participants in the intervention group attended on-site sessions involving video-guided exercise with staff assistance, whereas participants in the control group attended weekly face-to-face educational lectures regarding knee and low back pain. The trial was conducted at a community-based exercise facility in Iruma, Japan, and the study-related procedures took place at Saitama Medical University. The study period was from June 10, 2025, to October 17, 2025.

### Participants

The study participants were community-dwelling adults living in the vicinity of the Karada Ugoki Kaifuku Center, Iruma. All participants who were randomized to either the intervention or control group and who contributed any valid daily-life walking data were eligible for inclusion. We included older adults aged 65 years and older who were able to walk independently and access the facility and experienced mild to moderate knee and/or low back pain, with pain intensity rated as less than 8 on a visual analog scale (VAS) from 0 to 10. The exclusion criteria were inability to walk independently, severe joint pain (VAS score ≥8), presence of medical conditions such as neurological or respiratory disease that made participation in the exercise program inappropriate as judged by a physician, and difficulty with decision-making because of dementia or impaired consciousness.

### Sample Size

The sample size was determined based on the primary outcomes of the parent trial, specifically the Roland-Morris Disability Questionnaire and the Western Ontario and McMaster Universities Arthritis Index. A sample size of 20 participants per group was calculated to provide 90% power to detect the expected effect based on the mean intergroup difference and SD reported in a similar randomized controlled trial [1,2,25,26]. To allow for attrition and missing outcome data, the total recruitment target was set at 60 participants, comprising 30 participants per group. A separate sample size calculation was not performed for this secondary analysis.

### Randomization and Blinding

Participants were randomly assigned to the intervention or control group in a 1:1 ratio using a computer-generated random sequence prepared by the Department of Biostatistics, Saitama Medical University Graduate School of Medicine. Specifically, the sequence was generated using the SAS software (PROC PLAN statement; SAS Institute) with a fixed random seed, and randomization was performed using permuted blocks with a block size of 6. Participant enrollment was conducted by the second author (T Arai), whereas group assignment was implemented by the Biostatistics Group at Saitama Medical University and was performed independently of the intervention team. Blinding of participants and intervention staff was not feasible due to the nature of the interventions; however, the outcome assessors for performance-based measures were blinded, and questionnaire outcomes were self-reported.

### Baseline Measures

Baseline characteristics for which data were collected in the parent trial included demographics (age and sex), anthropometrics and body composition (height, body weight, BMI, and skeletal muscle index), physical performance (maximal grip strength, standing time on one leg, and comfortable and maximal walking speed), pain and symptom measures (the low back and knee pain VAS score, Roland-Morris Disability Questionnaire score, and Western Ontario and McMaster Universities Arthritis Index subscale and total scores), and health-related questionnaires (25-question Geriatric Locomotive Function Scale, Kihon Checklist, Geriatric Depression Scale, and EQ-5D index).

### Interventions

Participants in the intervention group received an on-site exercise program delivered through the Curves personalized exercise support system, which aimed to alleviate knee and low back pain through individually prescribed video programs with staff present to support participation and ensure safety. The program consisted of 21 distinct exercises comprising 12 stretching exercises and 9 muscle strengthening exercises. Stretching was performed to the maximal comfortable range of motion, with each of the 12 positions held for 30 seconds. Regarding muscle strengthening, upper-limb and lower-limb exercises used body weight resistance or auxiliary equipment such as elastic bands and a rubber ball, with intensity adjusted to a “somewhat hard” perceived exertion level. Strengthening exercises were performed for 30 seconds each at a speed based on the participants’ subjective intensity. The planned frequency and duration were 2 sessions per week for 12 weeks. The specific content and progression rules were updated based on the participants’ exercise proficiency and visit frequency according to age. Participants initially started with 15 exercises and progressed to performing the full 21-exercise program after 4 weeks. Staff monitored participant responses and implemented safety protocols, including discontinuing exercise for a specific body part if acute pain occurred and adjusting movements to a pain-free range of motion if pain was present. The control group received on-site educational lectures on knee and low back pain once weekly during the intervention period, and these participants were offered access to the intervention program after the completion of follow-up assessments.

### Secondary Analysis Outcomes

This secondary analysis focused on daily-life walking outcomes derived from an ankle-worn activity monitor, WAlkX (Sanka). We analyzed daily activity volume outcomes, representing the daily accumulation of distance, steps, active energy expenditure, and activities graded at 3 or more metabolic equivalents of task (METs). In addition, we analyzed gait characteristics, representing the daily average values for walking speed, cadence, and step length. Participants were asked to wear the device continuously unless it caused discomfort.

The device was worn continuously during waking hours throughout the monitoring period, including during the on-site sessions; participants were not asked to remove it. Because the supervised sessions comprised video-guided multicomponent exercises performed in place rather than continuous overground walking, in-session activity did not generate the gait bouts detected by the daily-life walking algorithm and did not contribute to the step count or gait parameters. Therefore, the reported outcomes reflect habitual out-of-session daily-life walking.

The preintervention window was the 7 days starting 1 day after device placement. The postintervention window was the 7 days immediately preceding each participant’s last observed monitoring day, capturing late-program or end-of-program daily-life walking. The timing of the last monitoring day relative to the scheduled program end date varied across participants (in most cases, it clustered near program completion, with earlier cessation of monitoring in some participants), reflecting differences in device return and wear adherence.

### Adherence, Protocol Deviations, and Safety

Adherence was quantified as the number of sessions attended and the percentage of prescribed sessions completed during the intervention period, with adequate adherence for the per-protocol analysis defined as completion of 80% to 120% of the prescribed sessions. Protocol deviations and adverse events were monitored throughout the study.

### Statistical Analysis

For this secondary analysis, we use the term “modified intention-to-treat” (mITT) analysis because it included all randomized participants who contributed at least one valid daily-life walking observation in both windows rather than all randomized participants. Daily-life walking outcomes were analyzed using repeated-measure linear mixed-effects models (LMMs) incorporating all available daily observations from participants who contributed at least one valid observation in both the pre- and postintervention windows. The LMMs were prespecified based on the repeated-measure data structure rather than selected from a normality test of the outcome distributions. Daily observations were extracted from 2 planned 7-day windows anchored to the monitoring period. The preintervention window was defined as the 7 days starting 1 day after participants began wearing the device (ie, excluding the first day to avoid potential reactivity or partial wear effects). The postintervention window was defined as the 7 days immediately preceding the last observed monitoring day for each participant. Days with no recorded step counts (steps=0) were treated as missing daily walking observations and were excluded from model fitting. As a sensitivity analysis, we conducted a per-protocol set (PPS) analysis based on the trial ledger; we included participants classified as PPS eligible. Intervention effects were estimated using repeated-measure LMMs, with the primary parameter of interest being the group × phase interaction term, interpreted as a difference-in-differences (DID) estimate.

For each outcome, we fitted a repeated-measure LMM including fixed effects for group, phase, day in window, and the group × phase interaction, with a participant-specific random intercept.

Model estimates are reported as regression coefficients with 95% CIs. Models were fitted using restricted maximum likelihood in Python (version 3.14.0; Python Software Foundation) using statsmodels (version 0.14.6) with Pandas (version 3.0.1), NumPy (version 2.4.2), and SciPy (version 1.17.1).

### Ethical Considerations

The study was conducted in accordance with the principles of the Declaration of Helsinki and the relevant Japanese ethical guidelines. It is reported in accordance with the CONSORT (Consolidated Standards of Reporting Trials) 2010 statement. The protocol was approved by the Saitama Medical University Institutional Review Board (approval 2025-004; May 24, 2025). The trial was registered in the University Hospital Medical Information Network Clinical Trials Registry (UMIN000057169) [27]. All participants provided informed consent prior to enrollment.

## Results

### Overview

The baseline characteristic data of participants in the parent trial are summarized in Table 1. Overall, 60 participants were assessed for eligibility in the present study; however, 1 withdrew after providing consent. Thus, 59 participants were randomized, with 30 assigned to the intervention group and 29 assigned to the control group. The flow of study participants is summarized in Figure 1 (CONSORT flow diagram).

**Table 1.** Parent trial participant characteristics^a^.

Variables	Intervention group (n=30)	Control group (n=28)	Intergroup difference	Welch *P* value	SMD^b^
Sex (female), n (%)	13 (43.3)	11 (39.3)	4	.76	0.08
Age (y), mean (SD)	73.7 (5.4)	74.9 (5.2)	−1.2	.39	−0.23
Height (cm), mean (SD)	161.5 (10.1)	161.2 (8.3)	0.3	.90	0.03
Body weight (kg), mean (SD)	59.1 (10.0)	61.6 (11.2)	−2.5	.37	−0.24
BMI (kg/m^2^), mean (SD)	22.6 (3.0)	23.6 (3.1)	−1.0	.22	−0.33
Skeletal muscle index (kg/m^2^), mean (SD)	7.1 (1.0)	7.3 (1.1)	−0.2	.48	−0.19
Maximal grip strength (kg), mean (SD)	30.3 (7.0)	29.3 (8.0)	1.0	.62	0.13
Standing time on one leg (eyes open; s), mean (SD)	46.2 (41.3)	31.1 (30.8)	15.1	.12	0.41
Comfortable walking speed (m/s), mean (SD)	1.2 (0.2)	1.2 (0.3)	0.0	>.99	0.00
Maximal walking speed (m/s), mean (SD)	1.7 (0.3)	1.7 (0.3)	0.0	>.99	0.00
Low back pain VAS^c^ score (0-10), mean (SD)	2.7 (2.4)	3.5 (2.8)	−0.8	.25	−0.31
RDQ^d^ total score (range 0-24), mean (SD)	3.0 (3.2)	4.2 (4.8)	−1.2	.27	−0.30
Knee pain VAS score (0-10), mean (SD)	2.7 (2.2)	3.7 (2.7)	−1.0	.13	−0.41
WOMAC^e^ pain subscale score (range 0-20), mean (SD)	3.0 (2.3)	4.2 (3.9)	−1.2	.16	−0.38
WOMAC stiffness subscale score (range 0-8), mean (SD)	1.1 (1.4)	2.0 (1.8)	−0.9	.04	−0.56
WOMAC physical function subscale score (range 0-68), mean (SD)	7.2 (8.6)	11.6 (11.4)	−4.4	.11	−0.44
WOMAC total score (range 0-96), mean (SD)	11.3 (10.8)	17.8 (15.5)	−6.5	.07	−0.49
LOCOMO-25^f^ total score (range 0-100), mean (SD)	10.0 (6.6)	16.0 (10.9)	−6.0	*.*02	−0.67
Kihon Checklist total score (range 0-25), mean (SD)	4.5 (3.0)	5.5 (2.9)	−1.0	.20	−0.34
GDS^g^ total score (range 0-15), mean (SD)	3.3 (3.2)	3.0 (2.7)	0.3	.70	0.10
EQ-5D index score, mean (SD)	0.9 (0.1)	0.8 (0.2)	0.1	.02	0.64

a Intergroup difference = intervention − control. The Pearson chi-square test was used for sex; the Welch *t* test was used for continuous variables. Baseline characteristics are shown for randomized participants with an available baseline assessment (intervention: n=30; control: n=28); 1 control participant lacked baseline data.

b SMD: standardized mean difference.

c VAS: visual analog scale.

d RDQ: Roland-Morris Disability Questionnaire.

e WOMAC: Western Ontario and McMaster Universities Arthritis Index.

f LOCOMO-25: 25-question Geriatric Locomotive Function Scale.

g GDS: Geriatric Depression Scale.

**Figure 1.**
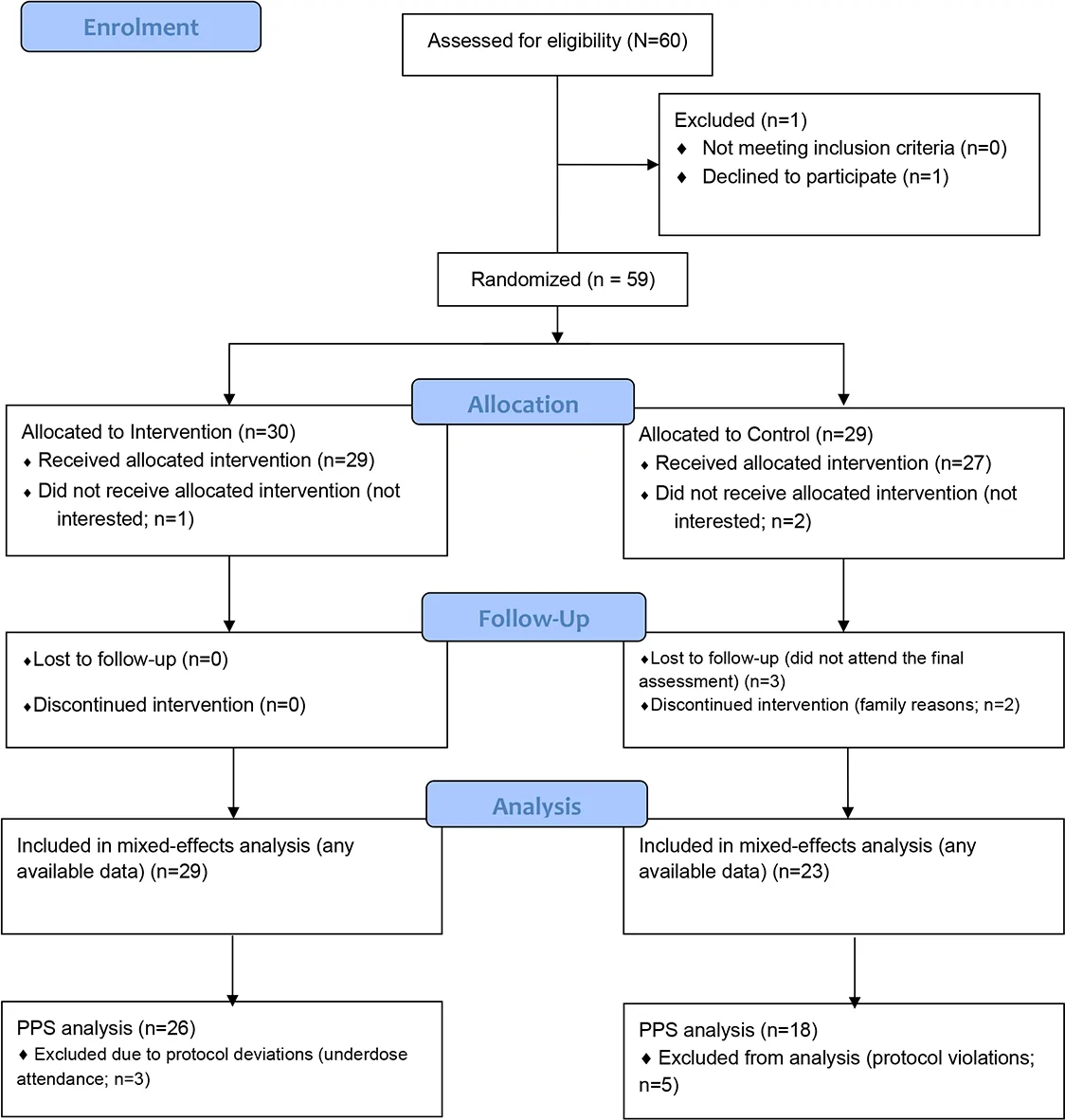
CONSORT (Consolidated Standards of Reporting Trials) flow diagram of participant allocation, follow-up, and details of analysis sets, including the final modified intention-to-treat and per-protocol set (PPS) mixed-effects samples after the planned missing data handling for daily walking observations.

A total of 56 participants contributed at least one valid daily observation within the planned 7-day pre- and/or postintervention windows. Of these, 54 participants (intervention: n=29; control: n=25) were mITT eligible (ie, they contributed at least one valid daily observation in both windows). Three randomized participants contributed no valid activity monitor data within the planned windows, and 2 further control participants had observations from only 1 window. Of the 54 mITT-eligible participants, 52 (intervention: n=29; control: n=23; 696 daily observations) contributed analyzable observations to the fitted mixed-effects models after treating days with no recorded steps (steps=0) as missing. The 2 remaining control participants had valid prewindow days but no analyzable (nonzero steps) postwindow days; therefore, they could not contribute a within-participant pretest-to-posttest contrast and were not estimable in the fitted models.

In the intervention group, the median number of sessions attended was 23 (IQR 21‐24) over the 12-week period. As a sensitivity analysis, a PPS was defined based on the trial ledger. This yielded 47 PPS-eligible participants (intervention: n=27; control: n=20), of whom 44 contributed at least one valid daily observation in both windows (intervention: n=26; control: n=18).

### Mixed-Effects Analysis Using All Available Data

In the mixed-effects models using all available daily observations, 52 participants contributed 696 daily observations across the 2 planned windows (before and after the intervention). The PPS sensitivity analysis contributed 44 participants and 595 observations.

In the mixed-effects models using 696 daily observations from 52 participants, the intervention was associated with a directional effect toward better gait quality, which was most clearly observed for step length. The DID estimate for daily-life step length was 2.39 cm (*P*=.04; Hedges *g*=0.493), whereas walking speed showed a smaller effect with borderline statistical significance (β=0.05 m/s; *P*=.06; Hedges *g*=0.496). Cadence did not differ clearly between the groups (β=1.26 steps per minute; *P*=.50). Daily steps and active energy expenditure also did not significantly differ between the groups (β=–51.0 steps per day and *P*=.92; β=4.84 kcal per day and *P*=.83). The effects of the intervention on daily distance and accumulated 3 or more MET activities were likewise null (β=0.08 km per day and *P*=.75; β=0.08 MET hours per day and *P*=.81; Table 2). As the gait quality outcomes were assessed alongside multiple related outcomes, these findings were interpreted as signals rather than as definitive confirmatory evidence.

**Table 2.** Results of the modified intention-to-treat analysis using mixed-effects models with all available daily observations.

Outcome variables	Participants, n	Observations, n	DID^a^ coefficient (SE; 95% CI)	*P* value	Hedges *g*^b^
Distance (km/d)	52	696	0.08 (0.26; −0.42 to 0.59)	.75	0.076
Steps (steps per d)	52	696	−51.00 (521.02; −1072.21 to 970.20)	.92	0.000
Acal^c^ (kcal per d)	52	696	4.84 (22.05; −38.38 to 48.06)	.83	0.100
MET^d^ h (MET h per d; ≥3 METs)	52	696	0.08 (0.32; −0.55 to 0.71)	.81	0.079
Speed (m/s)	52	696	0.05 (0.03; −0.00 to 0.10)	.06	0.496
Step length (cm)	52	696	2.39 (1.18; 0.09 to 4.70)	*.*04	0.493
Cadence (steps per min)	52	696	1.26 (1.88; −2.43 to 4.95)	.50	0.312

a DID: difference in differences. DID indicates the group × phase interaction.

b Standardized mean difference in change (after − before) with small-sample correction.

c Acal: activity-related calorie expenditure.

d MET: metabolic equivalent of task.

### PPS Analysis

The PPS sensitivity analysis included 44 participants who provided 595 daily observations. This analysis revealed the same directional pattern as observed with the primary model but with wider CIs. The DID estimate for step length was 1.95 cm (*P*=.13), whereas that for walking speed was 0.04 m/s (*P*=.14) and that for cadence was 1.03 steps per minute (*P*=.62). The effects of the intervention on daily steps and active energy expenditure remained null in the PPS analysis (β=–286.25 steps per day and *P*=.62; β=–8.88 kcal per day and *P*=.72). The effects on daily distance and accumulated 3 or more MET activities were also null (β=–0.01 km per day and *P*=.98; β=–0.09 MET hours per day and *P*=.80; Table 3).

**Table 3.** Results of the per-protocol analysis using mixed-effects models.

Outcome variables	Participants, n	Observations, n	DID^a^ coefficient (SE; 95% CI)	*P* value	Hedges *g*^b^
Distance (km/d)	44	595	−0.01 (0.28; −0.56 to 0.55)	.98	−0.015
Steps (steps per d)	44	595	−286.25 (576.25; −1415.70 to 843.19)	.62	−0.121
Acal^c^ (kcal per d)	44	595	−8.88 (24.67; −57.23 to 39.47)	.72	−0.095
MET^d^ h (MET h per d; ≥3 METs)	44	595	−0.09 (0.35; −0.78 to 0.60)	.80	−0.071
Speed (m/s)	44	595	0.04 (0.03; −0.01 to 0.10)	.14	0.368
Step length (cm)	44	595	1.95 (1.29; −0.59 to 4.48)	.13	0.365
Cadence (steps per min)	44	595	1.03 (2.08; −3.04 to 5.10)	.62	0.125

a DID: difference in differences. DID indicates the group × phase interaction.

b Standardized mean difference in change (after − before) with small-sample correction.

c Acal: activity-related calorie expenditure.

d MET: metabolic equivalent of task.

### Trajectory of Daily-Life Walking Outcomes

Figure 2 shows descriptive day-by-day trajectories for daily steps (Figure 2A) and daily walking speed (Figure 2B) from days 2 to 98 aligned to each participant’s day 1 (first recorded date). The daily walking speed trajectory showed a modest separation between the groups, emerging around the midintervention phase and persisting up to the end of follow-up, whereas the daily step trajectory did not show a similarly clear pattern. According to participation logs, the median program started on day 27 (IQR 26‐29) in the control group and day 29 (IQR 28‐30) in the intervention group. These figures are descriptive, and formal inference is provided by the mixed-effects models.

**Figure 2.**
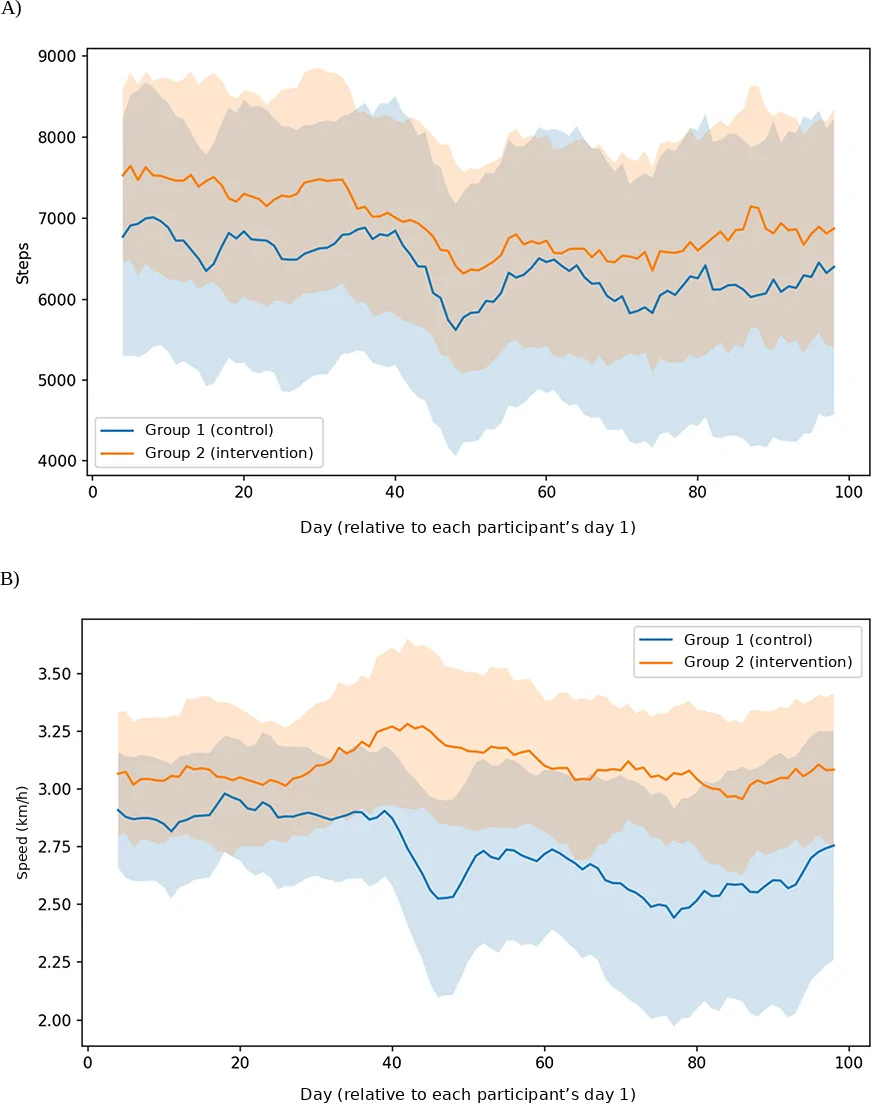
Daily steps and walking speed trajectories. (A)**. **Daily step trajectory (days 2‐98). Lines depict the 7-day rolling mean of the group’s daily mean step count, with each day indexed relative to each participant’s day 1 (first recorded date). Shaded bands indicate smoothed 95% CIs for each group’s daily mean (95% CI = mean ±1.96 × SE, also smoothed using a 7-day rolling mean). (B) Daily walking speed trajectory (days 2‐98). Lines depict the 7-day rolling mean of the group’s daily mean walking speed, with each day indexed relative to each participant’s day 1 (first recorded date). Shaded bands indicate smoothed 95% CIs around each group’s daily mean (mean ±1.96 × SE, also smoothed using a 7-day rolling mean). The figure is descriptive as formal inference is provided by the mixed-effects models.

### Adverse Events

One adverse event was reported in the control group, and no adverse events were reported in the intervention group.

## Discussion

Walking parameters, particularly walking speed and step length, are well-established indicators of adverse health outcomes such as mortality and disability [1,28]. There is accumulating evidence suggesting that these daily-life gait characteristics are also informative for health risk, including the risk of falls and frailty [20,22,29].

### Principal Findings

In this study, we examined whether a hybrid multicomponent training program influenced daily-life walking. In mixed-effects analyses using all available daily observations, the clearest effect of the intervention was observed on step length, whereas cadence showed no clear effect of the intervention and walking speed showed a smaller effect, with borderline statistical significance. Although the step length estimate is of a moderate effect size (Hedges *g* of approximately 0.49), the small sample size of this secondary analysis yielded a wide CI; therefore, whether the change reaches a minimal clinically important difference remains uncertain. Activity volume outcomes, including daily steps and active energy expenditure, were not clearly altered by the training program. Overall, the effect of the intervention was more consistently observed on gait quality than on activity volume.

In this study, the most consistent effect of the intervention was observed on step length, whereas changes in cadence were small and imprecise, particularly in the PPS analysis. This pattern suggests that any intervention-related change in daily-life walking quality may have been driven more by step length than by cadence. A previous study demonstrated that daily-life cadence was associated with ambient temperature and may be modulated by autonomic responses [30]. Such influences may attenuate intervention-related changes in cadence compared with more mechanically determined gait parameters such as step length [31]. Although these findings remain exploratory, a between-group difference in step length of approximately 2 cm is not negligible from a gait quality perspective in older adults.

In contrast to the consistent improvements observed in gait characteristics, effects on activity volume outcomes were less clear. The multicomponent program did not incorporate components designed to promote behavior change, such as goal setting, self-monitoring, or structured activity coaching. Moreover, the intervention was intentionally designed as a scalable, video-guided program delivered without continuous specialist supervision. Although this format supports scalability, it may make it more difficult to embed individualized, multifaceted behavior change support. Prior evidence similarly indicates that interventions focused on symptoms and physical function do not necessarily lead to increases in objectively measured physical activity [32]. When increasing activity volume is an explicit goal, exercise-based interventions may need to incorporate behavior change techniques [33,34].

The descriptive walking speed trajectory (Figure 2B) was presented to provide an intuitive representation of how intergroup differences may have evolved over time. According to the participation logs, program initiation clustered around days 26 to 30 (control group: median day 27, IQR 26‐29; intervention group: median day 29, IQR 28‐30), and the speed trajectory suggested modest visual separation between the groups from approximately day 40. This timing corresponds to approximately 2 weeks after initiation and is broadly consistent with that in exercise-based randomized trials for knee osteoarthritis, in which intergroup differences could be detected within approximately 2 weeks for pain and functional outcomes and within approximately 3 weeks for changes in walking speed [35,36]. Notably, the trajectory plot suggested a maintenance pattern in the intervention group rather than a marked improvement. The observed intergroup difference may therefore reflect prevention in the intervention group of the decline observed in the control group.

### Limitations

We observed some inconsistencies in statistical significance between the results of mixed-effects analyses using all available daily observations and those of the PPS sensitivity analyses for certain outcomes. Across multiple activity-related measures, including steps per day, daily walking distance, active energy expenditure, and MET-based indexes, no consistent intervention effect was observed. In the PPS set, the smaller sample size reduced statistical precision and widened CIs, making statistical significance less likely. However, the overall interpretation was consistent across analytic sets. Model residuals departed from normality for several outcomes, and in a distribution-robust sensitivity check, the one nominally significant outcome (step length) was no longer statistically significant; therefore, the findings are interpreted as suggestive.

This study has some limitations despite its strengths, such as objective, automatically derived free-living gait outcomes and comparison against an active control condition. The participants were recruited from a single setting and were required to attend sessions, which may limit generalizability. Additionally, this was a secondary analysis of activity monitor–derived outcomes, and the parent trial sample size was determined for symptom outcomes rather than specifically for the present free-living gait outcomes; accordingly, precision may have been limited for modest between-group differences. The cohort was clinically heterogeneous in baseline symptom burden; combined with the modest sample size of this secondary analysis, this may have increased between-participant variability and limited the precision of the between-group estimates. Participants who refused to wear the device or withdrew before outcome collection contributed no activity monitor data within the planned pre- and postintervention windows and, therefore, did not contribute data to the mixed-effects analyses of device-derived outcomes. The postwindow anchor was the last monitoring day rather than a fixed postcompletion assessment date, so the exact interval between intervention completion and the measured window differed between participants. The per-protocol definition may have also influenced PPS estimates, and the PPS findings should be interpreted as sensitivity analyses. Finally, we did not directly measure key mediators of change (eg, pain trajectories), and longer follow-up will be needed to assess the durability and downstream clinical outcomes.

### Practical Implications

This study has practical implications for scalable pain management services. Many exercise-based programs for knee and low back pain demonstrate benefits under professional supervision, yet widespread implementation is constrained by time and workforce. The current hybrid model of video-guided sessions with staff assistance may offer a feasible option that can be delivered at scale while potentially being consistently associated with a signal for daily-life gait quality. From a clinical perspective, the gait outcomes are clinically meaningful because gait speed is a robust indicator of overall health and ability to function and predicts adverse outcomes such as disability and mortality in older adults [28,37-39]. In the present study, the speed signal was modest and imprecise, whereas the step length signal was more consistent. Hence, clinical interpretation may focus primarily on gait quality rather than on activity volume while recognizing that even modest changes in physical performance measures may be clinically meaningful in older adults [40].

### Conclusions

A 12-week hybrid multicomponent program combining video-guided exercise with staff assistance may be associated with a suggestive signal for better daily-life walking quality, with the clearest effect observed for step length in older adults with knee and low back pain. However, given the attenuation in sensitivity analyses and the multiplicity of outcomes assessed, these findings should be regarded as exploratory.

## Supplementary material

10.2196/97414 Checklist 1 CONSORT checklist.

## Abbreviations

CONSORT: Consolidated Standards of Reporting Trials
DID: difference in differences
LMM: linear mixed-effects model
LOCOMO: Longitudinal Cohorts of Motor System Organ
MET: metabolic equivalent of task
mITT: modified intention to treat
PPS: per-protocol set
ROAD: Research on Osteoarthritis Against Disability
VAS: visual analog scale
