# Supplementary material for: Association Between a Hybrid Video-Guided Multicomponent Exercise Program With Staff Assistance and Free-Living Gait in Older Adults With Knee and Low Back Pain: Secondary Analysis of a Randomized Controlled Trial
Source: JMIR Aging. 2026 Oct 8;9:e97414. doi: 10.2196/97414 (PMC13649242; doi:10.2196/97414)
Supplement: Supplementary file 1 [file aging-v9-e97414-s001.docx]

**CONSORT 2010 Checklist of Information to Include When Reporting a Randomised Trial***

**Schulz KF, Altman DG, Moher D, for the CONSORT Group (2010). Ann Intern Med 152.*

**Manuscript:** Association of a Hybrid Video-guided Multicomponent Exercise Program with Staff Assistance and Free-living Gait in Older Adults with Knee and Low Back Pain: A Secondary Analysis of a Randomized Trial

**Authors:** Shuichi P Obuchi, Tomoyuki Arai, Hideaki Ishibashi, Takashi Amari, Hisashi Kawai

**Trial Registration:** UMIN000057169

*Note: Page numbers refer to the submitted manuscript (docx). Please verify against final formatted version.*

| **Section/Topic** | **Item No.** | **Checklist Item** | **Reported on Page No / Section** |
| --- | --- | --- | --- |
| **TITLE AND ABSTRACT** | | | |
|  | **1a** | Identification as a randomised trial in the title | Page 1. Title: "...A Secondary Analysis of a Randomized Trial" |
|  | **1b** | Structured summary of trial design, methods, results, and conclusions (for specific guidance see CONSORT for abstracts) | Page 2. Abstract: Background, Objectives, Methods, Results, Conclusions |
| **INTRODUCTION** | | | |
| **Background and objectives** | **2a** | Scientific background and explanation of rationale | Pages 3–4. Introduction section |
|  | **2b** | Specific objectives or hypotheses | Page 4. Introduction, final two paragraphs |
| **METHODS** | | | |
| **Trial design** | **3a** | Description of trial design (such as parallel, factorial) including allocation ratio | Page 4. Methods, Study Design and Setting: secondary analysis of a parallel-group RCT, 1:1 allocation |
|  | **3b** | Important changes to methods after trial commencement (such as eligibility criteria), with reasons | Page 7. Methods, Ethical Considerations: Trial protocol accessible via UMIN Clinical Trials Registry (UMIN000057169) at https://center6.umin.ac.jp/cgi-open-bin/ctr/ctr_view.cgi?recptno=R000065218 |
| **Participants** | **4a** | Eligibility criteria for participants | Page 4. Methods, Participants: aged ≥65, independent walking, VAS score <8. Exclusions listed. |
|  | **4b** | Settings and locations where the data were collected | Page 4. Methods, Study Design: Karada Ugoki Kaifuku Center, Iruma, Japan; Saitama Medical University. June 10 to October 17, 2025. |
| **Interventions** | **5** | The interventions for each group with sufficient details to allow replication, including how and when they were actually administered | Page 5. Methods, Interventions: Intervention—Curves video-guided system, 21 exercises (12 stretching, 9 strengthening), 2×/week for 12 weeks, staff-assisted. Control—weekly educational lectures. |
| **Outcomes** | **6a** | Completely defined pre-specified primary and secondary outcome measures, including how and when they were assessed | Page 5. Methods, Secondary Analysis Outcomes: daily-life walking from WalkX ankle monitor. Activity volume (distance, steps, Acal, and METs) and gait characteristics (speed, cadence, and step length). Two planned 7-day windows. |
|  | **6b** | Any changes to trial outcomes after the trial commenced, with reasons | Page 7. Methods, Ethical Considerations: Trial protocol accessible via UMIN Clinical Trials Registry (UMIN000057169) at https://center6.umin.ac.jp/cgi-open-bin/ctr/ctr_view.cgi?recptno=R000065218 |
| **Sample size** | **7a** | How sample size was determined | Page 4. Methods, Sample Size: n=20/group for 90% power based on parent trial (RDQ, WOMAC). Target 60 total for attrition. No separate calculation for secondary analysis. |
|  | **7b** | When applicable, explanation of any interim analyses and stopping guidelines | Not applicable. |
| **RANDOMISATION** | | | |
| **Sequence generation** | **8a** | Method used to generate the random allocation sequence | Page 4. Methods, Randomization and Blinding: SAS software (PROC PLAN) with fixed random seed. |
|  | **8b** | Type of randomisation; details of any restriction (such as blocking and block size) | Page 4. Methods, Randomization: 1:1 ratio, permuted blocks, block size of six. |
| **Allocation concealment mechanism** | **9** | Mechanism used to implement the random allocation sequence (such as sequentially numbered containers), describing any steps taken to conceal the sequence until interventions were assigned | Page 4. Methods, Randomization: sequence prepared by Dept. of Biostatistics, Saitama Medical University. Assignment by Biostatistics Group, independent of intervention team. |
| **Implementation** | **10** | Who generated the random allocation sequence, who enrolled participants, and who assigned participants to interventions | Page 4. Methods, Randomization: Sequence—Biostatistics dept. Enrolment—T.A. (second author). Assignment—Biostatistics Group, independently. |
| **BLINDING** | | | |
|  | **11a** | If done, who was blinded after assignment to interventions (for example, participants, care providers, those assessing outcomes) and how | Page 4. Methods, Randomization and Blinding: Participants and staff not blinded. Outcome assessors for performance-based measures blinded. Sensor-derived outcomes (this analysis) are objective/automated. |
|  | **11b** | If relevant, description of the similarity of interventions | Pages 4 and 5. Both groups on-site. Intervention: 2×/week exercise. Control: 1×/week lectures. Frequency and content differ. |
| **STATISTICAL METHODS** | | | |
|  | **12a** | Statistical methods used to compare groups for primary and secondary outcomes | Page 6. Methods, Statistical Analysis: Repeated-measures LMMs. Fixed: Group, Phase, DayInWindow, Group × Phase. Random: participant intercept. REML. Python 3.14.0, statsmodels 0.14.6. DID estimate. Hedges g. |
|  | **12b** | Methods for additional analyses, such as subgroup analyses and adjusted analyses | Page 6. Methods, Statistical Analysis: PPS sensitivity analysis (80–120% sessions). Same LMM. |
| **RESULTS** | | | |
| **Participant flow (a diagram is strongly recommended)** | **13a** | For each group, the numbers of participants who were randomly assigned, received intended treatment, and were analysed for the primary outcome | Page 8. Results: 60 assessed; 1 withdrew; 59 randomized (30 intervention, 29 control); 54 mITT-eligible (29 + 25) with 52 (29 + 23) contributing to fitted models.  Page 23, Figure 1: CONSORT flow diagram. |
|  | **13b** | For each group, losses and exclusions after randomisation, together with reasons | Pages 7-8. Results: 3 with no valid sensor data; 2 controls with only 1 phase.  Page 23, Figure 1: CONSORT flow diagram. |
| **Recruitment** | **14a** | Dates defining the periods of recruitment and follow-up | Page 4. Methods: June 10 to October 17, 2025. Pre-window: 7 days from day 2. Post-window: last 7 days. Program start ~day 27–29. |
|  | **14b** | Why the trial ended or was stopped | Trial completed as planned after the 12-week intervention period. |
| **Baseline data** | **15** | A table showing baseline demographic and clinical characteristics for each group | Pages 7-8. Table 1: age, sex, BMI, physical performance, VAS, RDQ, WOMAC, LOCOMO-25, Kihon Checklist, GDS, and EQ-5D with intergroup differences and SMDs. |
| **Numbers analysed** | **16** | For each group, number of participants (denominator) included in each analysis and whether the analysis was by original assigned groups | Pages 8-9. Results: mITT n=52 (696 observations). PPS n=44 (595 observations). mITT principle stated. |
| **Outcomes and estimation** | **17a** | For each primary and secondary outcome, results for each group, and the estimated effect size and its precision (such as 95% confidence interval) | Pages 9-10. Table 2 (mITT): DID, SE, 95% CI, P, Hedges g for 7 outcomes. Step length 2.39 cm (0.09-4.70, P=.042, g=0.493). Speed 0.05 m/s (-0.00-0.10, P=.063, g=0.496).  Page 12, Table 3 (PPS). |
|  | **17b** | For binary outcomes, presentation of both absolute and relative effect sizes is recommended | N/A—all outcomes continuous. Hedges g reported (Tables 2, 3). |
| **Ancillary analyses** | **18** | Results of any other analyses performed, including subgroup analyses and adjusted analyses, distinguishing pre-specified from exploratory | Page 10. PPS sensitivity analysis (Table 3).  Pages 10-11, Figure 2: descriptive day-by-day trajectories (steps, speed) with rolling means. |
| **Harms** | **19** | All important harms or unintended effects in each group (for specific guidance see CONSORT for harms) | Page 11. Results, Adverse Events: 1 adverse event in control group; none in intervention group. |
| **DISCUSSION** | | | |
| **Limitations** | **20** | Trial limitations, addressing sources of potential bias, imprecision, and, if relevant, multiplicity of analyses | Pages 11–13. Discussion, Limitations: single-site; secondary analysis powered for parent outcomes; PPS definition; no mediator measurement; no longer follow-up; multiplicity of outcomes. |
| **Generalisability** | **21** | Generalisability (external validity, applicability) of the trial findings | Pages 11–13. Discussion, Limitations and Practical Implications: single community (Iruma, Japan); required independent ambulation and facility access. |
| **Interpretation** | **22** | Interpretation consistent with results, balancing benefits and harms, and considering other relevant evidence | Pages 11–13. Discussion, Principal Findings: step length effect most consistent; cadence less responsive; intervention may prevent decline; comparison with prior RCTs [32, 33]; activity volume unchanged—lacks behavior change components [30, 31]. |
| **OTHER INFORMATION** | | | |
| **Registration** | **23** | Registration number and name of trial registry | Page 7. Methods, Ethical Considerations: UMIN000057169 |
| **Protocol** | **24** | Where the full trial protocol can be accessed, if available | Page 7. Methods, Ethical Considerations: Trial protocol accessible via UMIN Clinical Trials Registry (UMIN000057169) at https://center6.umin.ac.jp/cgi-open-bin/ctr/ctr_view.cgi?recptno=R000065218 |
| **Funding** | **25** | Sources of funding and other support (such as supply of drugs), role of funders | Pages 13–14. Funding: Curves Japan (grant, venue, intervention delivery). Funder had no role in data analysis, interpretation, or manuscript preparation. |
